# Use of the D-R Model to Define Trends in the Emergence of Ceftazidime-Resistant *Escherichia coli* in China

**DOI:** 10.1371/journal.pone.0027295

**Published:** 2011-12-06

**Authors:** Fan Ding, Dante S. Zarlenga, Yudong Ren, Guangxing Li, Jin Luan, Xiaofeng Ren

**Affiliations:** 1 College of Veterinary Medicine, Northeast Agricultural University, Harbin, China; 2 Animal Parasitic Diseases Laboratory, Agricultural Research Service, Beltsville, Maryland, United States of America; 3 Department of Computer, College of Engineering, Northeast Agricultural University, Harbin, China; 4 China's Armed Police Force Center for Disease Control, Lang Fa, Da Xing District, Beijing, China; University of Birmingham, United Kingdom

## Abstract

**Objective:**

To assess the efficacy of the D-R model for defining trends in the appearance of Ceftazidime-resistant *Escherichia coli*.

**Methods:**

Actual data related to the manifestation of Ceftazidime-resistant *E. coli* spanning years 1996–2009 were collected from the China National Knowledge Internet. These data originated from 430 publications encompassing 1004 citations of resistance. The GM(1,1) and the novel D-R models were used to fit current data and from this, predict trends in the appearance of the drug-resistant phenotype. The results were evaluated by *Relative Standard Error* (RSE), *Mean Absolute Deviation* (MAD) *and Mean Absolute Error* (MAE).

**Results:**

Results from the D-R model showed a rapid increase in the appearance of Ceftazidime-resistant *E. coli* in this region of the world. These results were considered accurate based upon the minor values calculated for RSE, MAD and MAE, and were equivalent to or better than those generated by the GM(1,1) model.

**Conclusion:**

The D-R model which was originally created to define trends in the transmission of swine viral diseases can be adapted to evaluating trends in the appearance of Ceftazidime-resistant *E. coli*. Using only a limited amount of data to initiate the study, our predictions closely mirrored the changes in drug resistance rates which showed a steady increase through 2005, a decrease between 2005 and 2008, and a dramatic inflection point and abrupt increase beginning in 2008. This is consistent with a resistance profile where changes in drug intervention temporarily delayed the upward trend in the appearance of the resistant phenotype; however, resistance quickly resumed its upward momentum in 2008 and this change was better predicted using the D-R model. Additional work is needed to determine if this pattern of “increase-control-increase” is indicative of Ceftazidime-resistant *E. coli* or can be generally ascribed to bacteria acquiring resistance to drugs in the absence of alternative intervention.

## Introduction

*Escherichia coli* is a Gram negative rod-shaped bacterium that is commonly found in the lower intestine of warm-blooded organisms. Although most *E. coli* strains are harmless, some such as serotype O157:H7, can cause serious food poisoning in humans [1], [2]. Symbiotic strains are part of the normal flora of the gut and can benefit their hosts by producing vitamin K_2_ [3], and by preventing pathogenic bacteria from establishing within the intestine [4], [5].

Harmful bacterial infections are usually treated with antibiotics. In contrast to Gram-positive organisms, resistance to antibiotics is more likely found among strains of *E. coli*. Ceftazidime is a third-generation cephalosporin antibiotic which has broad spectrum activity against both Gram-positive and Gram-negative bacteria; however, it is less effective in ridding Gram-positive microorganisms and therefore used mostly to treat Gram-negative infections in China.

Antibiotic resistance is a growing problem due to overuse in humans and in some cases overuse in the feed given to animals as a means to promote growth and weight gain [6]. This is particularly true for Ceftazidime in China. Evaluation of the appearance of Ceftazidime-resistant *E. coli* may provide guidance on the appropriate clinical use of this and other antibiotics. Herein, we used a novel algorithm, the D-R model and compared this to another algorithm, the Grey Model, GM(1,1), to simulate the resistance to Ceftazidime and to predict future trend lines for the appearance of this phenotype. In our studies, the D-R model showed fit and forecasting characteristics that equaled or bettered those generated by the GM(1,1) model. Importantly, the resistance of *E. coli* to this drug showed a pseudobiphasic response where a period of stable or decreasing resistance was followed by an abrupt increase or reemergence of the phenotype. These data and the D-R algorithm may provide useful and more general references for controlled application of antibiotics clinically.

## Materials and Methods

### Data collection

Data on Ceftazidime-resistant *E. coli* in China between 1996 and 2009 were collected from the China National Knowledge Internet (CNKI). The keywords for the search included *E. coli*, Ceftazidime and/or drug resistance. Generally, the data were derived from hospitals and institutes of more than thirty provinces. The rules for data collection were as follows: 1) recorded drug resistance was restricted to *E. coli*; 2) Ceftazidime-resistance rates in *E. coli* were to be from literature sources only; 3) data sets had to be defined by resistant strain number and statistical year, and; 4) data had to pass manual verification as not being duplicated within the data set. If collected resistance data extended over consecutive years, these data were partitioned equally among the years spanned. The total number of reports of drug-resistant *E. coli* per year/total number of reports of *E. coli* infections per year = normalized annual drug-resistance rate. The statistical analysis was performed by trend χ2 test using SPSS13.0 software.

### Calculations and model fitting

Time series prediction is the process by which future values of a system can be forecast based on past and current data points. If all the information about a system is available, it is considered a White system. If no information exists, it is a Black system. Those systems described by limited available data are by definition Grey systems. One of the most common algorithms used today for forecasting future trends is the GM(1,1) model from grey system theory [7] which requires only a few historical and current data points to estimate the future behavior of an unknown system. Based upon grey system theory and the GM(1,1) model, the D-R model [8] was first developed to predict future trends in H1N1 cases in mainland China. The D-R model gives less consideration to micro-environmental factors. It establishes upper and lower limits of the prediction intervals and accounts for dominating long or short term effects by incorporating self-adapting parameters into the equation. Comparing the two models, we found that the D-R model had better predictive value than the GM(1,1) model for the H1N1 data set.

We decided to expand the usability of the D-R model and test its predictive value on forecasting the spread of Ceftazidime-resistant *E. coli*. As such, the D-R and GM(1,1) models were used to calculate the Ceftazidime-resistance rates of *E. coli* extending from 1996 to 2009 in China. Comparisons between the actual values and calculated values were analyzed using the *Relative standard error* (RSE), *Mean absolute deviation* (MAD) and *Mean Absolute Error* (MAE) to test the accuracy of the fit. These relationships are defined by the equations below (10):
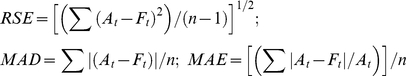
Where A_t_ = the actual drug resistance data; F_t_ = fitted or calculated drug resistance data; and n = sample number.

### Future predictions in drug resistance

Once the best model was identified, the actual Ceftazidime-resistance rates for *E. coli* between 1996 and 2009 in China were used to predict future Ceftazidime-resistance rates for *E. coli* between 2009 and 2012 using that model. The forecasting abilities of the D-R model were compared to those generated by the GM(1,1) model. Also, in order to assess the importance of incorporating current data and the ability of the D-R model to adapt to inflections points in the data, we evaluated the predictive character of the D-R model using information from four different time frames; 1996–1998, 1996–2000, 1996–2004 and 1996–2008. These time frames were chosen because each preceded mini peaks in the actual drug-resistance rates found in 1997, 2000, 2004 and 2008.

## Results

### Statistical analysis

Analysis was performed on 1004 pieces of data defining Ceftazidime-resistance rates in *E.coli* The normalized drug-resistance rate/year evaluated by χ^2^ test (*(trendχ^2^ test)*) had a p<0.01 indicating that the Ceftazidime-resistance rates for *E. coli* significantly increased during the period 1996–2009.

### Fitting of D-R and GM(1,1) models

**Figure 1:**
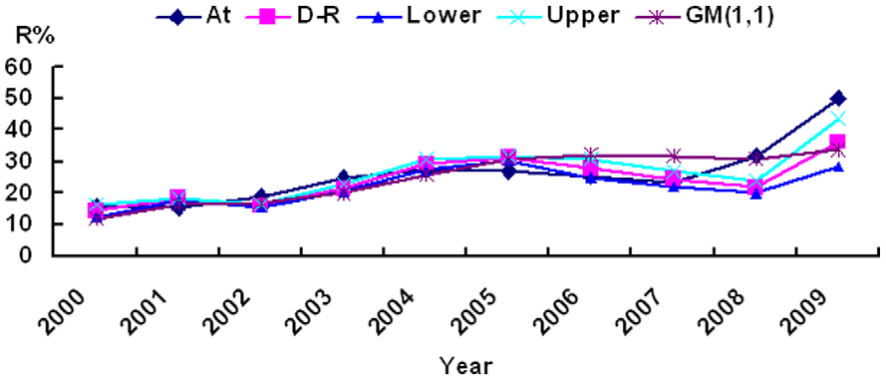
Observed and predicted rates of *E. coli* resistance to Ceftazidime using the D-R and GM(1,1) models. At = actual data; D-R = rates forecasted using the D-R model; Upper and Lower = the limits of the D-R model; GM(1.1) = rates forecasted using the GM model.

The relationship between the actual Ceftazidime-resistance rates for *E. coli* and the predicted values were analyzed with D-R and GM(1,1) models (Figure 1; Table 1). Based on the calculated data and the actual data, the RSE, MAD and MAE values were determined and summarized in Table 2. The values based on the D-R algorithm were noticeably more in line with the actual data than those generated using the GM(1,1) model indicating that D-R algorithm was as good as if not better than the GM(1,1) model for predicting changes to the trend line of *E. coli* resistance to Ceftazidime.

**Table 1:** Actual and predicted Ceftazidime-resistance rates between 1996∼2009 in China.

Year	At	D-R	Lower	Upper	GM(1,1)
1996	5.71	-	-	-	-
1997	13.08	-	-	-	-
1998	11.07	-	-	-	-
1999	12.75	-	-	-	-
2000	16.00	14.19	12.28	16.12	11.96
2001	15.29	18.11	17.77	18.44	16.27
2002	19.07	15.87	15.14	16.60	16.75
2003	24.78	21.57	20.38	22.77	19.92
2004	27.51	29.04	27.42	30.67	25.78
2005	26.84	31.02	30.19	31.85	30.53
2006	25.06	27.82	25.00	30.65	32.31
2007	23.39	24.17	21.60	26.75	31.98
2008	31.66	21.87	19.79	23.95	30.74
2009	50.24	36.33	28.24	43.74	33.79

At = actual drug resistance rate; D-R = calculated rates using the D-R model; Lower = lower limits of the D-R model; Upper = upper limits of the D-R model; GM(1,1) = calculated rates using the GM(1,1). No data is designated by “-”.

**Table 2:** Calculated values for RSE, MAD and MAE from rate curves generated with the D-R and GM(1,1) models.

Model	RSE	MAD	MAE
**D-R**	6.23	4.40	0.15
**GM(1,1)**	7.15	5.08	0.18

Calculated values were based on the actual Ceftazidime-resistance rates between 1996–2009 in China.

### Forecasting future rates of *E. coli* Ceftazidime-resistance using the D-R model

**Figure 2:**
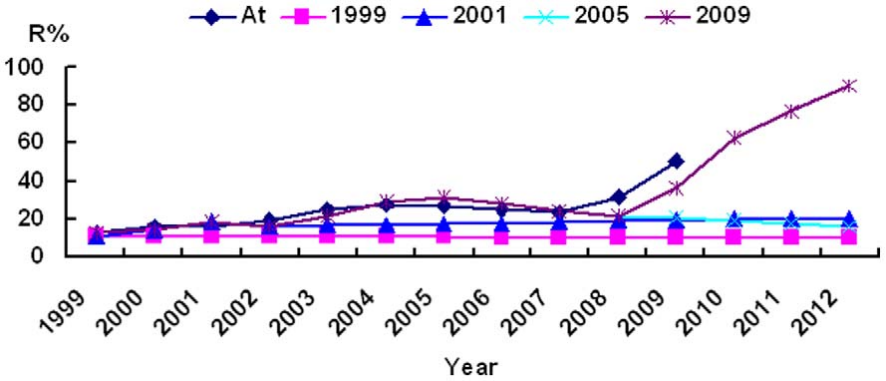
Use of the D-R model to forecast changes in drug resistance rates. Forecasting changes in the rate of appearance of Ceftazidime-resistant *E. coli* was performed over several time frames. Based upon data obtained from years 1996–1999, 1996–2000, 1996–2004 and 1996–2008, rates were predicted beginning with years 1999, 2001, 2005, and 2009, respectively. The year 1999 = predicted rates of infection in 1999–2012 based on data obtained from 1996–1998; 2001 = predicted rates of infection in 2001–2012 based on data obtained from 1996–2000; 2005 = predicted rates of infection in 2005–2012 based on the data obtained from 1996–2004; 2009 = predicted future rates of infection in 2009–2012 based on the data obtained from 1996–2008. For each time period analyzed, the obtained data were fitted to the model and graphed in addition to the predicted rates of infection.

Based upon calculations of Ceftazidime-resistance rates between 1996 and 2009 using the D-R model, the Ceftazidime-resistance rates for 2010, 2011 and 2012 were projected to be 63.20%, 76.56% and 90.08%, respectively (Table 3, Figure 2), provided there are no changes in drug intervention. However, using any other time intervals to predict future trends i.e. 1996–1998, 1996–2000, and 1996–2004, the results showed no appreciable changes in the rates beyond 20% inasmuch as none of these included the recent inflection point observed in the years 2007–2008. This difference in predictive value reflects the importance of recent data in forecasting and the ability of the D-R model to better incorporate these types of inflection points than the GM(1,1) model.

**Table 3:** D-R generated Ceftazidime-resistance rates beginning with years 1999, 2001, 2005, and 2009.

Year	A_t_	1999F_t_(R%)	2001F_t_(R%)	2005F_t_(R%)	2009F_t_(R%)
1996	5.71				
1997	13.08				
1998	11.07	____			
1999	12.75	10.73	10.73	12.38	12.38
2000	16.00	10.96	14.19	14.19	14.19
2001	15.29	10.76	18.11	18.11	18.11
2002	19.07	10.66	15.87	15.87	15.87
2003	24.78	10.58	16.45	21.57	21.57
2004	27.51	10.48	16.86	29.04	29.04
2005	26.84	10.38	17.31	31.02	31.02
2006	25.06	10.29	17.76	27.82	27.82
2007	23.39	10.20	18.19	24.17	24.17
2008	31.66	10.12	18.61	21.87	21.87
2009	50.24	10.03	19.03	20.34	36.33
2010		9.95	19.44	18.84	63.20
2011		9.86	19.84	17.37	76.56
2012		9.78	20.23	15.93	90.08

At = actual drug resistance rate; 1999 Ft(R%) = predicted infection rates between 1999–2012 based on the actual data from 1996–1998; 2001Ft(R%) = predicted infection rates between 2001–2012 based on the actual data from 1996–2000; 2005Ft(R%) = predicted infection rates between 2005–2012 based on the actual data from 1996–2004; 2009Ft(R%) = predicted infection rates between 2009–2012 based on the actual data from 1996–2008. The numbers above the underlined values were calculated and fitted to the model; the numbers below the underlined values were predicted based upon each fitted curve.

## Discussion

In China, Ceftazidime is among the most commonly prescribed antibiotics for treating E. coli infections. Unfortunately, its misuse and overuse has resulted in Ceftazidime-resistant organisms. Surveillance for the drug-resistant phenotype is therefore important and can be an effective method for controlling the escalation and spread of these bacteria.

Accuracy indices defined by RSE, MAD and MAE are extensively used when fitting data, where smaller values are indicative of more accurate associations between calculated data sets [8]–[10]. In this study, the RSE, MAD, ARE values using the D-R model coincided well with actual values indicating the potential use of this model for predictive purposes. Upon examination of the actual data, we found that the drug-resistance rates generally increased between 1996 and 2005 after which they slightly decreased then resumed an upward trend but at a faster rate beginning in 2008. Four mini peaks were found in 1997, 2000, 2004 and 2008. Coinciding with each peak year, literature citations were found which argued the importance of decreasing the rate of drug application as a means to control resistance. This relationship is circumstantial at best; however, Ceftazidime-resistance always seemed to increase at a later point in time and after the cautionary notes on attenuating usage waned.

Using the D-R model and based on the data obtained between 1996–1998, 1996–2000, 1996–2004 and 1996–2008, we predicted the future trends between 1999–2012, 2001–2012, 2005–2012 and 2009–2012, respectively (Table 3, Figure 2). From Figure 2, we can see that before 2008, had constrained drug treatment or combination drug therapy been used, the drug resistance rate may have been more easily controlled. In contrast, invoking such measures after 2008 may have had little to no effect in altering the rate of Ceftazidime-resistance going forward.

Clearly, more strict control of administering antibiotics is needed to decrease the spread and occurrence of drug-resistant bacteria. Our results showed that in 2009, the rate of reported drug-resistance cases exceeded 30%. In the absence of any material change in treatment, we estimate that in 2010, the rate will exceed 60% implying that combination drug therapy should be reinforced. Furthermore, if the current trends are not altered by a change in treatment regiments, we anticipate that by 2011, the rate could exceed 75%. The statistical data on the rate of appearance of Ceftazidime-resistant *E. coli* was 50.24% in 2009. This is consistent with our predictive results indicating the utility of D-R model in this analysis.

In this study, we simulated the currently available data on drug-resistance rates of *E. coli* using D-R and GM(1,1) models. The Ceftazidime-resistant *E. coli* data from 1996–2009 in China were subjected to trend χ^2^ test, and the results showed that Ceftazidime-resistance increased annually where the reported rate reached 30% and 50% in 2008 and 2009, respectively. The caveat to this study as well as any that are based on preexisting data, is that it is predicated only upon reports of patients who sought medical help and were treated without response. It is difficult to account for those who did not seek medical attention, or the many that were treated with predictable results and thus never reported. Clearly, more extensive epidemiological studies are necessary to confirm the findings reported in this paper. Nonetheless, our data show with incontrovertible evidence that the trend in resistance experienced a dramatic inflection point to the upside in 2008 and that this trend was predicted by the D-R model. With this in mind, we must now pay particular attention to the precautionary limits set by the Ministry of Health (MOH) of the People' Republic of China. The regulations defined by the MOH state: 1) when drug resistance to a target bacterium is over 30%, early warning information should be reported to all medical personnel; 2) when resistance exceeds 40%, the drug should be used cautiously and empirically; 3) if resistance climbs above 50%, use of the drug should be first verified with a drug sensitivity test and; 4) when resistance exceeds 75%, use of the drug should be stopped and reintroduction should be based upon large scale surveillance studies demonstrating a reduction in the drug resistant phenotype to manageable levels (http://www.moh.gov.cn/publicfiles/business/htmlfiles/mohyzs/s3585/200903/39723.htm). To this end, we highly recommend that with the increase in the rate of infection observed herein, more extensive surveillance studies be conducted to monitor resistance in the Chinese population before it reaches unmanageable levels.

As with any forecast model under development, there are caveats. Under- and over- estimation of future trends is by and large the biggest hurdle. For this reason, the D-R model was designed to provide upper and lower limits to the forecasted trend line. In both the H1N1 study [8] and the evaluation of Ceftazidime-resistant *E. coli* presented here, the upper and lower limits to the trend line were never breached suggesting that such limits are important in encompassing variables that could affect forecasting future changes.

In conclusion, we used the most up-to-date data on the rate of Ceftazidime-resistance in *E. coli* in China to calculate future rate changes using the new D-R model and the classic GM(1,1) model. Our results showed that during a period of time when resistance exhibited a slow but increasing trend as in most early periods of resistance modeling, both algorithms were equally effective in their abilities to predict future rate changes. However, when strong inflection points occurred as observed in 2008, the (GM)1,1 model was unable to adequately mirror the actual rates that followed whereas, the D-R model more accurately accounted for these rapid bursts in resistance.
